# Evidence from *in vivo *31-phosphorus magnetic resonance spectroscopy phosphodiesters that exhaled ethane is a biomarker of cerebral *n*-3 polyunsaturated fatty acid peroxidation in humans

**DOI:** 10.1186/1471-244X-8-S1-S2

**Published:** 2008-04-17

**Authors:** Basant K Puri, Serena J Counsell, Brian M Ross, Gavin Hamilton, Marcelo G Bustos, Ian H Treasaden

**Affiliations:** 1 MRI Unit, MRC Clinical Sciences Centre, Imaging Sciences Department, Imperial College London, Hammersmith Hospital, Du Cane Road, London W12 0HS, UK; 2 Division of Medical Sciences, Northern Ontario School of Medicine, Lakehead University, Room MS 3002, 955 Oliver Road, Thunder Bay, Ontario, Canada P7B 5E1, and Department of Chemistry, Lakehead University, and Public Health Program, Lakehead University, Thunder Bay, Ont., Canada P7B 5E1; 3 Department of Radiology, UCSD School of Medicine, 408 Dickinson Street, San Diego, CA 92103-8226, USA; 4 Three Bridges Medium Secure Unit, West London Mental Health NHS Trust, Uxbridge Road, Southall, Middlesex UB1 3EU, UK

## Abstract

**Background:**

This study tested the hypothesis that exhaled ethane is a biomarker of cerebral *n*-3 polyunsaturated fatty acid peroxidation in humans. Ethane is released specifically following peroxidation of *n*-3 polyunsaturated fatty acids. We reasoned that the cerebral source of ethane would be the docosahexaenoic acid component of membrane phospholipids. Breakdown of the latter also releases phosphorylated polar head groups, giving rise to glycerophosphorylcholine and glycerophosphorylethanolamine, which can be measured from the 31-phosphorus neurospectroscopy phosphodiester peak. Schizophrenia patients were chosen because of evidence of increased free radical-mediated damage and cerebral lipid peroxidation in this disorder.

**Methods:**

Samples of alveolar air were obtained from eight patients and ethane was analyzed and quantified by gas chromatography and mass spectrometry (*m*/*z *= 30). Cerebral 31-phosphorus spectra were obtained from the same patients at a magnetic field strength of 1.5 T using an image-selected *in vivo *spectroscopy sequence (TR = 10 s; 64 signal averages localized on a 70 × 70 × 70 mm^3 ^voxel). The quantification of the 31-phosphorus signals using prior knowledge was carried out in the temporal domain after truncating the first 1.92 ms of the signal to remove the broad component present in the 31-phosphorus spectra.

**Results:**

The ethane and phosphodiester levels, expressed as a percentage of the total 31-phosphorus signal, were positively and significantly correlated (*r*_*s *_= 0.714, *p *< 0.05).

**Conclusion:**

Our results support the hypothesis that the measurement of exhaled ethane levels indexes cerebral *n*-3 lipid peroxidation. From a practical viewpoint, if human cerebral *n*-3 polyunsaturated fatty acid catabolism can be measured by ethane in expired breath, this would be more convenient than determining the area of the 31-phosphorus neurospectroscopy phosphodiester peak.

## Background

Dioxygen (diatomic molecular oxygen), O_2_, is a toxic mutagenic gas, notwithstanding our dependence on O_2_-dependent electron-transport chains; we survive because of the presence of protective antioxidant defences [1]. Indeed, cellular reactive oxygen species such as superoxide radicals, O_2_^·-^, hydrogen peroxide, H_2_O_2_, and hydroxyl radicals, HO^·^, which are highly unstable oxygen species possessing reactive unpaired electrons, are generated during endogenous aerobic metabolism and in response to exogenous toxic challenges [2,3]. Since the living human brain normally has a high oxygen consumption and has a high lipid content, including oxyradical-sensitive polyunsaturated fatty acids (PUFAs), brain cell membranes are particularly vulnerable to free radical-mediated damage; under physiological conditions the potential for such damage is kept in check by the antioxidant defence system, which contains the critical antioxidant enzymes superoxide dismutase (SOD; E.C. 1.15.1.6), catalase (CAT; E.C. 1.11.1.6) and glutathione peroxidase (GSH-Px; E.C. 1.11.1.9) [4,5]. Peroxidative degradation is particularly marked in cerebral inner mitochondrial membrane lipids, owing to the fact that most cellular oxygen in the brain is used for terminal electron acceptance in oxidative phosphorylation [6,7]. SOD catalyzes the dismutation of O_2_^·- ^to H_2_O_2_, which is then converted into water and molecular oxygen by reduction by GSH-Px, in conjunction with the conversion of glutathione into glutathione disulfide, and separately by CAT.

The study of evolution of the volatile hydrocarbon ethane was suggested as a means to detect and monitor levels of lipid peroxidation following the finding that homogenates of mouse brain gave off ethane gas during the process of cerebral lipid peroxidation (measured by the formation of malonaldehyde in the 2-thiobarbituric acid reaction) [8]. The time courses of lipid peroxidation and ethane evolution both proceeded essentially linearly from zero in the brain homogenates, with no time lag between the two. The addition of α-tocopherol, a free radical-trapping agent which blocks lipid peroxidation [9-11], at baseline completely prevented ethane formation, but if added instead after two hours, by which time lipid peroxidation had occurred, did not have a major effect on the subsequent formation of ethane. Further *in vitro *studies have shown that ethane is released specifically following peroxidation of *n*-3 (and *not n*-6) PUFAs, a class which includes the long-chain PUFAs eicosapentaenoic acid (EPA) and docosahexaenoic acid (DHA) [12,13]. Cell culture investigations support the hypothesis that ethane is an accurate indicator of *n*-3 fatty acid oxidation [14,15], while in a rodent study of the effects of dietary fatty acid intervention, it was reported that after being fed *n*-3 long-chain PUFA-rich cod liver oil, there was a linear increase in exhaled ethane over a period of three hours, compared with no increase in the exhalation of ethane in rats fed a low *n*-3 long-chain PUFA diet [16]. Therefore, measurement of exhaled ethane has been put forward as a putative measure of *n*-3 PUFA peroxidation in humans, particularly in the brain, for example in children suffering from attention-deficit hyperactivity disorder [17]. However, to date there have been no *in vivo *humans studies demonstrating that exhaled ethane is indeed a biomarker of cerebral *n*-3 PUFA peroxidation.

In attempting to provide such evidence, two aspects need to be addressed. First, a cohort of human subjects is required in whom there is increased cerebral *n*-3 PUFA peroxidation. Second, a known non-invasive method must be found which indexes the breakdown of cerebral *n*-3 PUFAs, so that its results can be directly compared with exhaled ethane levels. We examine each issue in turn.

It is clearly unethical to promote free radical damage, and therefore increased cerebral lipid peroxidation, in a cohort of human subjects. However, there are several converging lines of evidence pointing to free radical-mediated damage and perturbation of the body's defences against such damage in patients with the brain disorder schizophrenia. Erythrocyte antioxidant enzyme activity has been reported to be altered in chronic schizophrenia [18], with, in general, raised SOD activity [19-22], low or normal GSH-Px activity [21-23], and low CAT activity [22,24], which indicate decreased protection against oxidative injury, which could lead to membrane lipid peroxidation [18]. Finally, raised levels of membrane lipid peroxidation products have also been reported in schizophrenia, in both plasma [18,25,26] and cerebrospinal fluid [27,28]. Therefore it is appropriate to study a cohort of chronic medicated schizophrenia patients.

The remaining issue in investigating cerebral *n*-3 PUFA peroxidation in humans is to choose an appropriate non-invasive technique with which to compare the results of exhaled ethane levels in this patient group. If the source of ethane from the brain is *n*-3 PUFA peroxidation, then this must primarily be of DHA attached to the *sn*-2 position of neuronal and glial cell membrane phospholipids, and of intracellular organelle membrane phospholipids. Breakdown of such membrane phospholipids would release the phosphorylated polar head groups from the *sn*-3 phospholipid position, including phosphorylcholine and phosphorylethanolamine. Glycerophosphorylcholine and glycerophosphorylethanolamine, which are on their catabolic pathways [29], have been assigned to the phosphodiester (PDE) peak obtained from the non-invasive technique of 31-phosphorus nuclear magnetic resonance [30]. In a canine 31-phosphorus nuclear magnetic resonance study of the brain, the PDE peak was found to account for approximately 38 per cent of the overall signal; the figure for humans is the same [31]. A further analysis of the 31-phosphorus spectrum of a deproteinized methanol:HCl canine brain extract carried out at 144 MHz showed three resonances in the PDE region, at -0.9, -0.8, and 0.14 ppm: the resonance at -0.8 ppm had a p*K*_*a *_of 9.5, which is characteristic of the ethanolamine moiety, and coresonated and comigrated with glycerophosphorylethanolamine; the resonance at 0.14 ppm was not titratable and coresonated with glycerophosphorylcholine; the resonance at -0.9 ppm disappeared when the pH was lowered to 8.5 [31].

Therefore the technique we chose was 31-phosphorus neurospectroscopy, with the aim of testing the hypothesis that the ethane levels in alveolar air from chronic medicated schizophrenia patients correlate positively with the PDE signal from the same subjects.

## Methods

### Subjects

Eight male patients with a diagnosis of schizophrenia according to DSM-IV-TR [32] and aged between 28 and 61 years (mean age 41.1 years, standard deviation 10.8 years) were studied. All the patients suffered from chronic schizophrenia and were being treated with antipsychotic medication. The study was carried out according to the Declaration of Helsinki. The patients gave written informed consent. The study was approved by the local research ethics committee.

### Exhalant analysis

Each subject was asked to exhale through a disposable sterile mouthpiece into a syringe (Markes International Ltd., UK) in one long breath, until they were no longer able to exhale any further. This enabled alveolar (end expired) air to be collected from the lungs. The apparatus was designed in such a way that the same volume of end-expired air was collected from each patient. The air sample was then injected into an automated thermal desorption tube packed with carbotrap 300 (Perkin-Elmer, UK) via a sodium sulfate drying cartridge (International Sorbent Technology, UK). The air samples were analyzed using a Perkin-Elmer autosystem XL equipped with a turbo mass spectrometer. The automated thermal desorption tubes were desorbed onto the cold trap at 320°C, with the cold trap temperature being held at 5°C. The trap was then rapidly heated to 350°C and the liberated volatiles injected onto a 30 m × 0.32 mm PLOT GQ column (Perkin-Elmer, UK) with helium gas at 2 ml min^-1^. The oven was set at 45°C for 10 min and ramped at 14°C min^-1 ^to 200°C at which temperature it was held for 120 s. Ethane (C_2_H_6_) was eluted at 2.6 min and identified and quantified by mass spectrometry at an *m*/*z *value of 30 by comparison with a standard curve (0–60 pmol) constructed from a C1–C6 alkane standard mix (Supelco, UK).

For the ethane assay, variability and stability data were obtained using a group of 10 controls tested five days in a row with five tubes per test day. Inter-assay variability (as (standard deviation)/mean × 100%) was 17% and intra-assay variability was 10%. The method used was thermal desorption which is a very good way of collecting and immobilizing gases. The gas levels can reduce on the tube owing to chemical instability and simple desorption and diffusion. For the former ethane is a chemically stable molecule but desorption can occur. This was tested by introducing standards in air onto the tubes and testing at various times thereafter. It was found that after one week tubes retained 97% ethane, while retention was 95% after two weeks, and 90% after one month. Therefore the level diminishes over time, but slowly. Our samples were analyzed within one week of collection.

### *In vivo *spectroscopy

Cerebral 31-phosphorus magnetic resonance spectroscopy data were obtained using a 1.5 T Marconi Eclipse system (Marconi Medical Systems, Cleveland, Ohio) with a birdcage quadrature head coil dual-tuned to proton (^1^H, 64 MHz) and ^31^P (26 MHz). T_1_-weighted magnetic resonance images were acquired for spectral localization. Spectra were obtained using an image-selected *in vivo *spectroscopy sequence (ISIS) with a repetition time of 10 s with 64 signal averages localized on a 70 × 70 × 70 mm^3 ^voxel. Owing to the low abundance of ^31^P compared with ^1^H, the maximum size voxel was used to collect signal from the brain and thus maximize the signal-to-noise ratio.

All spectral analyses were carried out by a single observer (GH). The seven sets of peaks characteristically identifiable in the spectrum from a normal human brain were identified: in order of decreasing chemical shift, these peaks were assigned to phosphomonoesters, inorganic phosphate, phosphodiesters, phosphocreatine and gamma-, alpha- and beta-nucleotide triphosphate. The quantification of the ^31^P signals using prior knowledge was carried out in the time domain using the AMARES algorithm [33] included in the MRUI software program [34]. The first 1.92 ms of the signal was truncated to remove the broad component present in the ^31^P spectra and allow initial analysis of the narrow components listed above using *a priori *knowledge in the AMARES algorithm [35,36]. For each patient, the ratio of PDE to the total area under all seven sets of peaks was calculated and then multiplied by 100 to give the percentage PDE.

### Statistical analyses

Statistical analyses were carried out using the SPSS version 12 statistics program (SPSS Inc., Chicago).

## Results

**Figure 1:**
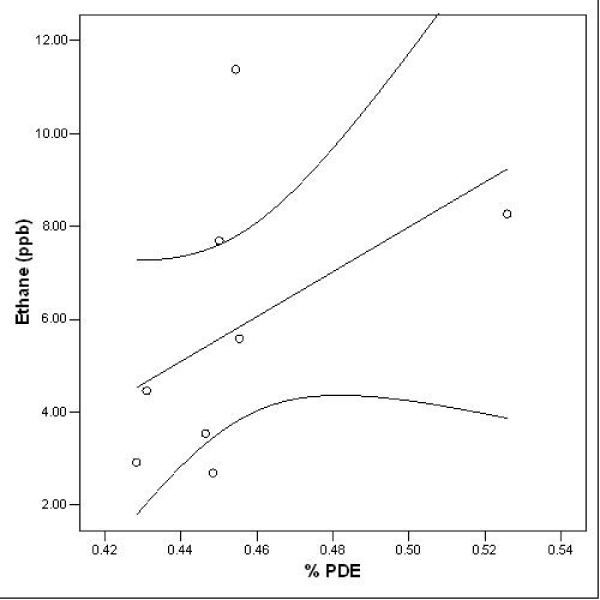
Levels of ethane (in ppb) in the expired breath of patients with schizophrenia plotted against their cerebral percentage PDE values, together with the straight line of best fit. The 95 per cent confidence interval for this regression line is also shown.

Since the percentage PDE values showed a marked deviation from gaussian expected values on a normal Q-Q plot, and gave a Kolmogorov-Smirnov statistic of 0.37, corresponding to a significant deviation from normality (*df *= 8, *p *< 0.05), a non-parametric measure of correlation was calculated between ethane levels and the corresponding percentage PDE values. These two variables showed a significant positive correlation (*r*_*s *_= 0.714, *p *< 0.05). The data, together with the straight line of best fit and its 95 per cent confidence interval, are shown in Figure 1.

## Discussion

In this first study of this type, we have found evidence of a positive correlation between levels of ethane in expired alveolar breath in human subjects and cerebral levels of phosphodiesters, which lends support to our hypothesis. The correlation coefficient between the two variables is high, at over 0.7. We would not expect a perfect correlation, since the long-chain PUFA at the *sn*-2 position of membrane phospholipids is not always DHA, but often arachidonic acid; ethane is not a catabolic metabolite of arachidonic acid. Furthermore, while either choline or ethanolamine, both of which are indexed by the PDE spectroscopy peak, often constitutes the polar head group at the *sn*-3 position of membrane phospholipids, this head group may also be inositol or serine, neither of which is known to be indexed by PDE. This might also explain why there is a negative intercept value on the ordinate in Fig. 1 at a percentage PDE value of zero; another contribution to this is likely to be experimental statistical error. Interestingly, if our hypothesis were true, then we might expect the regression line of best fit to pass through the origin. A reanalysis with this value leads to an even more significant and positive correlation (*r*_*s *_= 0.8, *p *< 0.01), with a majority of the experimentally determined data points continuing to lie within the 95 per cent confidence interval of the mean.

From a practical viewpoint, when studying human cerebral *n*-3 PUFA catabolism, it would clearly be more convenient, if possible, to measure ethane in expired breath than to determine the level of PDE. Taking a breath sample is quicker, easier and cheaper than carrying out 31-phosphorus neurospectroscopy. Moreover, magnetic resonance scanning is contraindicated in certain subjects, for example because of claustrophobia or safety reasons relating to the presence of certain types of implants. Furthermore, there are some patients who find it difficult to stay still for long enough to acquire meaningful data in a magnetic resonance scanner. An example is children with attention-deficit hyperactivity disorder. The prediction by the fatty acid model of attention-deficit hyperactivity disorder [37] that there might be an increase in cerebral phospholipid breakdown in this disorder was difficult to test directly using magnetic resonance spectroscopy, but a breath test investigation did indeed demonstrate raised levels of ethane in such children [17].

## Conclusion

The evidence from our study would appear to be consistent with the hypothesis that exhaled ethane levels index cerebral *n*-3 polyunsaturated fatty acid peroxidation, although further studies are required.

## Competing interests

The authors declare that they have no competing interests.

## Authors' contributions

All the authors made substantial contributions to the design and conception of the study. SJC and BKP were involved in data collection. BMR, GH, IHT and BKP analyzed the data. All authors were involved in the interpretation of the data. All the authors have been involved in drafting and revising the manuscript and have read and approved the final manuscript.
